# Supplementary material for: Non-Destructive Banana Ripeness Detection Using Shallow and Deep Learning: A Systematic Review
Source: Sensors (Basel). 2023 Jan 9;23(2):738. doi: 10.3390/s23020738 (PMC9866092; doi:10.3390/s23020738)
Supplement: Supplementary file 1 [file sensors-23-00738-s001.zip › sensors-2096932-supplementary.pdf]

Table S1. Collection data from the detection of banana ripeness studies

Authors	Types of banana samples	Varieties of banana categories	Wavelength/spectrum	Measurement tool	Camera /Sensor	Color space	Features	Classification stages	No. of data samples	Methods	Results (Accuracy & coefficient of determination R^2)
(Mendoza and Aguilera [43], (2004)	Fingers by hand, single batch	Musa cavendish	Visible	Colorimeter Hunter Lab with CIE standard illuminant D65, Digital refractometer	Fluorescent lamps, Digital camera	RGB	L*, a*, b*; BSA; NBS; texture feature	**7 stages-** green, green to traces of yellow, more green than yellow, more yellow than green, green tip, yellow, all yellow, yellow, flecked with brown	127	**Feature selection method-** Sequential forward selection (SFS) **Classifier:** Discriminant analysis (DA)	Classification Accuracy using DA-98%
(Mazen and Nashat [6], 2019a)	Fingers	Egyptian's species	Visible	NA	Digital camera	RGB and HSV	color, BSA, texture feature	**4 stages-** unripe (green banana), yellowish green, mid-ripe, overripe	300	ANN, SVM, Naïve Bayes classifier, KNN, Decision tree	ANN- 97.75 %, SVM -96.6%, Naïve Bayes classifier-95.5%, KNN-95.5%, Decision tree-97.75%
(S. E. Adebayo et al. [18], 2017)	Fingers	Not reported	532, 660, 785, 830, 1060nm	DA, universal testing machine, and Digital refractometer	LLBI, CCD	Laser, CCD	Chlorophyll, Elasticity, SSC	**6 stages (2-7)-** all green, green with some yellow, yellow with some green, yellow with few green, all yellow with some brown patches	270	ANN	- Chlorophyll prediction (532nm) = 0.949 **(R2)**<br>- Elastic modulus, SSC (785, 830nm) = 0.862, 0.867 **(R2)**<br>- ANN classification (830nm)- 95.5%
(Adebayo et al. [17], 2016)	Fingers	Musa Cavendish	532, 660, 785, 830, 1060nm	DA, universal testing machine, and Digital refractometer	LLBI, CCD	Laser, CCD	Elasticity, SSC, chlorophyll	**6 stages (2-7)-** all green, green with some yellow, yellow with some green, yellow with few green, all yellow with some brown patches	270	ANN	Accuracy- 97.53% (830, 1060)
(Saranya, Srinivasan, and Kumar, [9], 2021)	Fingers	Egyptian's species	Visible	NA	Digital camera	RGB	NA	**3 stages-** Ripe, partially ripe, ripen and overripe	273	CNN model (Transfer learning)-VGGNet 16, ResNet50)	- **Actual Dataset:** Accuracy-94.93%, Validation -87.5%<br>- **Augmented dataset:** Accuracy-96.14%, Validation -92.85%
(Ramadhan et al. n.d., [19])	Fingers	Musa Cavendish	Visible	NA	Digital camera	RGB	NA	**4 stages-** Unripe, Almost-Ripe, ripe Through Ripe	300	CNN	71.95%.

(Zhuang et al., [20] 2019)	Fingers	Musa Cavendish	Visible	NA	Camera, CCD	HSV, L*a*b*, CIE	Peel color, local textual, local shape (top, middle, tip) using UP-LBP, HOG	**4 stages-** all-green, mostly yellow with some or a little bit of green, all yellow, mostly yellow with some or many brown spots	441	Naïve Bayes, LDA, SVM	**Naïve Bayes, LDA, SVM-**<br>- color feature: 99.2% ,99.2%, 100%, and 99.2%<br>-local texture feature: 92.6%, 86.8%, and 93.4%<br>- local shape features: 84.3%, 83.5%, and 82.6%
(Sabilla et al., [21], 2019)	Fingers	other species	Visible	NA	Camera	RGB	Banana peel and images	**7 Category-** Ambon, Candi, Emas, Kepok, Raja, Santen, Susu	**Augmented data-** 5,193	KNN, SVM, Decision Tree	- KNN and DT-99.1% in terms of detecting the category of banana<br>-SVM-96.6%
(Hou et al., [22],2015)	Fingers	Musa Cavendish	Visible	portable tri-stimulus colorimeter (stalk, middle and tip)	Spectrometer	Spectrometer image	L*, a*, b*	**7 stages-** green, green to traces of yellow, more green than yellow, more yellow than green, green tip and yellow, all yellow, yellow, flecked with brown	210	SVM	-Radial-based kernel: 96.5%<br>-Linear-based kernel: 95.0%
(Adebayo et al., [35], 2017)	Fingers	Musa Cavendish	532, 660, 785, 830, 1060nm	ΔA meter, TA. XT Plus Texture Analyze	LLBI, CCD	CCD, laser	Chlorophyll, Elasticity, SSC	**6 stages (2-7)-** all green, green with some yellow, yellow with some green, yellow with few green, all yellow with some brown patches	270	SVM	92.5%
(Cho and Koseki, [10],2021)	Fingers	Musa Cavendish	250mm	Table-Top Universal testing Instruments using a cylindrical probe, refractometer, and colorimeter	Smartphone with 2-LED	HSV, L*a*b*, YUV	Firmness, pH, ratio pulp to peel	**3 stages-** Unripe, Ripe, Overripe	440	ANN	**R2-**<br>- Firmness: 0.97, TSS-0.96, pH - 0.60, ratio pulp to peel - 0.64
(Zhang et al., [23], 2018)	Fingers	Musa Cavendish	Visible	NA	Fluorescent lamps, Digital camera	RGB	color, shape, texture of the banana, local, and global features	**7** stages and **14** stages	197	CNN	**-7 ripening stages:** accuracy- 94.4%, precision-95.6%, Recall -92.3%<br>**-14 ripening stages:** Accuracy- 92.3 %, Precision- 93.5%, Recall - 93.1%
(Kipli et al., [38], 2018)	Fingers by hand	Not reported	Visible	NA	Smartphone	Global cloud vision	Color Dominant Extraction	**3 stages-** Unripe, Ripe, Overripe	338	J48 classification algorithm	Classification accuracy (training size of 30, 338)- 87.10%, 96.15%
(Mohamedon et al., [41], 2021)	Mixed	Not reported	Visible	NA	Smartphone	RGB	Color features	**3 stages-** Unripe, Ripe, Overripe	571	CNN using transfer learning	Accuracy- 98.25%

(Saragih and Emanuel, [44], 2021)	Fingers and fingers by hand	Egyptian species and not reported *	Visible	NA	Smartphone	RGB	RGB, HSVL, L*a*b*, texture	**4 stages-** unripe/green, yellowish - green, mid ripen, overripe	436	MobileNet V2, NASNet Mobile(Pretrained model)	Accuracy- Mobile Net V2 - 96.18%, NASNetMobile- 90.84%
(Altaf et al., [37], 2020)	Fingers by hand	Not reported	Visible	NA	Wireless sensor	Sensor	Temperature, humidity level, ethylene concentration, carbon dioxide level	**6 stages-** natural green, light green with light yellow, yellow with light green, yellow with brown spots, full yellow, and yellow with green ends.	8 samples	ANN	Overall accuracy- 96.78 %
(Chen et al., [40], 2018)	Bunch	Musa Cavendish	Visible	NA	Digital camera, Electronic-nose using DAQ, E-nose/Camera	RGB	aroma, color	**4 Stages-** unripe, half-ripe, fully ripe, overripe	**Sensor-** 105 measurements (7 days), **Peel color changes-** 105 samples	-PC +KNN<br>-PCA+SVM<br>-LDA + KNN<br>-LDA + SVM	**-Using camera (Feature=3)-** PCA +KNN= 99.05%, PCA+SVM= 97.14%, LDA + KNN= 99.05%, LDA + SVM= 94.29%<br>**-Using E-nose (Feature=7)-** PCA +KNN= 98.10%, PCA+SVM= 95.24%, LDA + KNN = 90.48%, LDA + SVM= 86.67%<br>**-Using E-nose/Camera (Feature=10)-** 100% (from all models)
(Zhu and Spachos, [24], 2021)	Fingers	Not reported	Visible	NA	Camera	HSV	HSV, texture	**3 Stages-** un-ripened, ripened, and over-ripened	**Actual data-** 150, **After augmentation-** 1000	**Classifier I-** KNN, RF, NB and SVM **Classifier 2-** YOLO v3 model (transfer learning)	-SVM-98.5% (I classifier-higher)<br>-YOLO v3 model- 85.7% (mid-ripped and well-ripped- classifier II<br>-Overall accuracy- 96.4%
(Mueez, [25], 2020)	Fingers	Egyptian's species	Visible	NA	Raspberry Pi 4 model B with sonar fitted, Camera	HSV	color of a peel	**4 stages-** green, yellowish-green, mid-ripen, overripen	273	KNN	-Actual data: 85%<br>-Augmented data: 97.75%
(Suthagar et al., [11], 2021)	Fingers	Not reported	Visible	MQ-3, MQ-6, MQ-8, MQ-135 sensors	An electronic nose, Arduino Board, LCD, Exhauster fan, and an array of sensors, Camera image	RGB	color and shape	**3 Stages-** UR-un-ripe stage; R&UR-neither ripe nor un-ripe stage; R-ripen stage.	24	An electronic nose, image processing technique, and manual	Accuracy -94.44%
(Zhu and Spachos,	Fingers	Not reported	Visible	NA	Camera	HSV	HSV, texture	**3 stages-** Un-ripped, ripped, overtripped	**Actual -** 150	SVM and YOLOv3 models	**Accuracy-(** Overall accuracy -96.4%)

[26], 2020)								**2 stages (2 layers)**- mid-ripped, well ripped	**Augmented**- 650		-SVM classifier-I: 98.5%<br>-YOLOv3 model-classifier II: 89.5%
(Maimunah et al. [27])	Fingers	Musa Cavendish (Ambon)	Visible	NA	Digital Camera, LCD	HSV	RGB, HSVL, L*a*b*, texture	**3 Stages** - stage 2, stage 3, stage 4 (maturity classes)	105	Naïve Bayes, neural network classifier	**Accuracy-**<br>-Naïve Bayes classifier I): 90.58%<br>-Neural network model-classifier II: 95.24%
(Iswari, Wella, and Ranny, [28], 2017)	Fingers	Musa Cavendish	Visible	Refractomezhuter	Digital Camera	RGB	3 colors, number of dots (yellow, green, brown), no of dots on a banana skin, sweetness level	**3 categories-** Banana, apple, melon	42	KNN	Graphical results
(Sanaeifar et al., [36], 2014)	Fingers	Musa Cavendish	Visible	MQ-3, MQ-6, MQ-135, MQ-131 sensors	Electronic nose	Sensors	3 colors and number of dots (yellow, green, brown), no of dots on a banana skin, sweetness level	**7 stages-** green, light green, half green and half yellow, more yellow than green, yellow with green tips, full yellow, and yellow with brown spots.	15	PCA, SIMCA, LDA, and SVM	**Accuracy-**<br>- SVM: 98.66%<br>- SIMCA: 92%<br>- LDA: 97.6%
(Rodrigues et al., [42], 2021)	Mixed	Not reported	Visible	NA	Camera	RGB	NA	**2 stages**- ripe and unripe	NA	VGG, VGG-16	Graphical results
(Marimuthu and Mohamed Mansoor Roomi, [39], 2017)	Fingers by hand	Musa Cavendish	Visible	NA	Digital Camera	RGB, HSV	pH, NBA	**3 stages**- ripe, overripe, unripe	**Augmented data-** 3108	FMCBR	Overall model accuracy -93.11%
(Zulkifli et al., [4], 2019)	Fingers	Musa Cavendish	658nm	Colorimeter, Digital refractometer	LLBI, CCD	CCD row data	L*a*b*, firmness, TSS, pH	**6 stages (2- 7)-** all green, green with some yellow, yellow with some green, yellow with few green, all yellow with some brown patches<br>**2 groups-** unripe, ripe	480	LDA	Overall model accuracy- 94.2%
(Intaravanne, Sumriddetchkajorn, and Nukeaw, [32], 2012b)	Fingers	Musa Cavendish	365nm	NA	Smartphone, visible and Fluorescent spectral	RGB, ultraviolet light	White light ratio, ultraviolet light ratio	**3 Stages-** immature, ripe, overripe	10	NA	Graphical results

(Saadl et al., [30], 2009)	Fingers	Not reported	Visible	NA	Webcam	RGB	Color	**3 Stages-** unripe, ripe, overripe	60	ANN	Classify 25 samples correctly out of 28 samples(images)
(Taghoy and Villaverde, [31], 2018)	Fingers	Musa Cavendish	Visible	MQ3, DHT22 temperature sensor	Arduino Uno	NA	Temperature, ethylene gas	**3 Stages-** unripe, ripe, overripe	1	fuzzy logic approach (toolbox in MATLAB)	Linear regression(R2)- 0.94
(Intaravanne, Sumriddetchkajorn, and Nukeaw, [29], 2012a)	Fingers	Musa Cavendish	Visible	NA	Smartphone, visible and Fluorescent spectral	White light, ultraviolet light	White light, ultraviolet light ratio	**3 Stages-** immature, ripe, and overripe	10	NA	Graphical results
(Xie, Chu, and He, [33], 2018)	Fingers	Musa Cavendish	Near-infrared (380-1023 nm)	colorimeter, texture analyzer	Hyperspectral imaging	Hyperspectral imaging	L*a*b*, firmness	**2 Stages-** ripe, unripe	168	PLS-DA model -PC1 + PC2 -PC1 + PC3 -PC2 + PC3	**-R2 values-** 0.795(L∗), 0.972(a∗), 0.773 (b∗) and 0.760 (firmness)<br>- **RPD value-** 2.234(L*), 6.098(a*), 2.119 (b*) and 2.062, (firmness).<br>- First three PCs (ripe and unripe)- 98.95% of the variance (PC1: 84.83%, PC2:12.63% and PC3:1.49%)
(Quevedo et al., [3], 2008)	Fingers	Musa Cavendish	Visible	NA	Digital camera, Fluorescent spectral	RGB	fractal texture	NA	5	Fractal Fourier method	Graphical results
(Mohapatra, Shanmugasunda Ramadhan , and Malmathanraj, [5], 2017)	Fingers	Musa cavendish (Red)	Visible	Dielectric properties measurement system	Digital camera	RGB	capacitance, relative, permittivity, impedance, and admittance changing during the ripening process	**7 Grade/ Stages -** 1 to 7	140	Nearest neighbor, FCM	**Average precision accuracy (%)-**<br>-CLBP + FCM- 96.82 %<br>- LBP + FCM- 93.91<br>- NRLBP + FCM- 97.61 %<br>**Accuracy (nearest neighbor)-** Grade 1- 86 %, Grade 2- 86%, Grade 3-84 %, Grade 4- 84%, Grade 5- 86%, Grade 6- 84%, Grade 7-84%
(Vaviya et al. n.d., [34])	Fingers	Not reported	Visible	NA	Smartphone	RGB	color and size	**2 Stages-** Unripe-ripened using CaC2, Unripe-ripe naturally	**Augmented data-** 1000	CNN	97%

NA- Not available, RGB- Red Green Blue, HSV-Hue Saturation Value, CCD- Charged Coupled Device, LLBI- Laser light backscattering imaging, VNIR- Resonon, Inc. visible near-infrared Pika L hyperspectral imaging, SSC- Soluble solid content, TSS- Total soluble content, YUV- Luminance and chrominance, NBA- Normalized brown area, pH- Peak hue, CNN- Convolutional Neural Network, ANN- Artificial Neural Network, BSA/cm^2- Brown spots percentage of the total area, NBS/cm^2- Number of brown spots per cm^2 of surface, UP-LBP- Local binary pattern Component Analysis, LDA- Linear Discriminant Analysis, KNN- K-Nearest Neighbor, DA- Delta Absorbance, PLS-DA- Partial least squares

discrimination analysis, DAQ- Data Acquisition Card, LED-Light emitting diode, FMCBR- Fuzzy modeling for the classification of Banana Ripeness, GUI-Graphical user interface, E-nose- Electronic nose, and LCD-Liquid crystal display, Sequential forward selection(SFS) method.

Table S2. Performance metrics for all included studies

Performance Metrics	Number of studies	Mean ± SD values
Accuracy	27	93.42 ± 5.24
R^2	3	0.85 ± 0.079
Graphical results	5	-

Table S3. Graphical representation studies for banana ripeness detection

Author (Year)	Graphical representation
Iswari, Wella, and Ranny, [28], (2017)	Application-based output (no numeric values)
Rodrigues et al., [42], (2021)	Probabilistic object identification-based output
Intaravanne et al., [29], (2012)	Image-based output
Intaravanne et al., [32], (2012)	Image-based output
Quevedo et al., [3], (2008) *****	Image-based output

Provided the graphical representation with fractal dimension (FD_h) value from 2.02 to 2.20.

Table S4. Following scoring, a scheme has been used for the quality assessment in this systematic review (* represented the scoring detailed information of the table)

Capturing device score	1(reported), 0 (Not reported/in completed information)
Number of samples (median thresholds)	1(above the threshold value), otherwise 0
Number of banana stages (mode thresholds)	1(above the threshold value), otherwise 0
Result score (Accuracy/correlation) (median thresholds)	1(above the threshold value), otherwise 0

*The capturing device score is considered as 1 if the detailed information of the device is mentioned in the studies. For the "number of samples", the score is 1 if it is more than the median threshold value and otherwise 0; similarly for the field "result score". In the case of "number of banana stages", the mode threshold value was used and the quality assessment of the studies was performed between the total score of 0 and 4 (if the value is between 0 and 2, it is considered as weak, 3 is considered fair and if the score > 4 it is considered as a strong study).

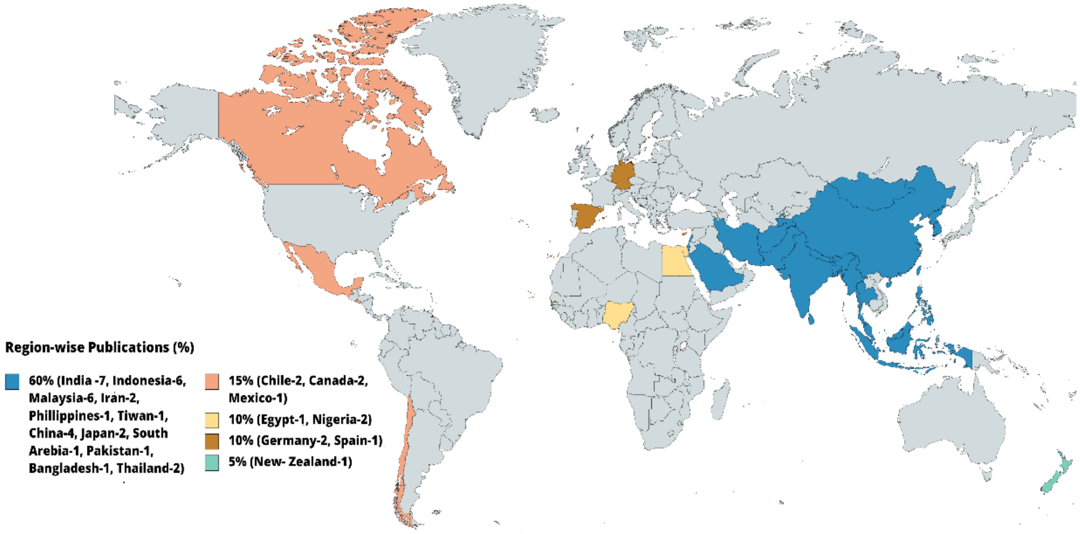

Figure S1. Region-wise publication based on author's affiliation

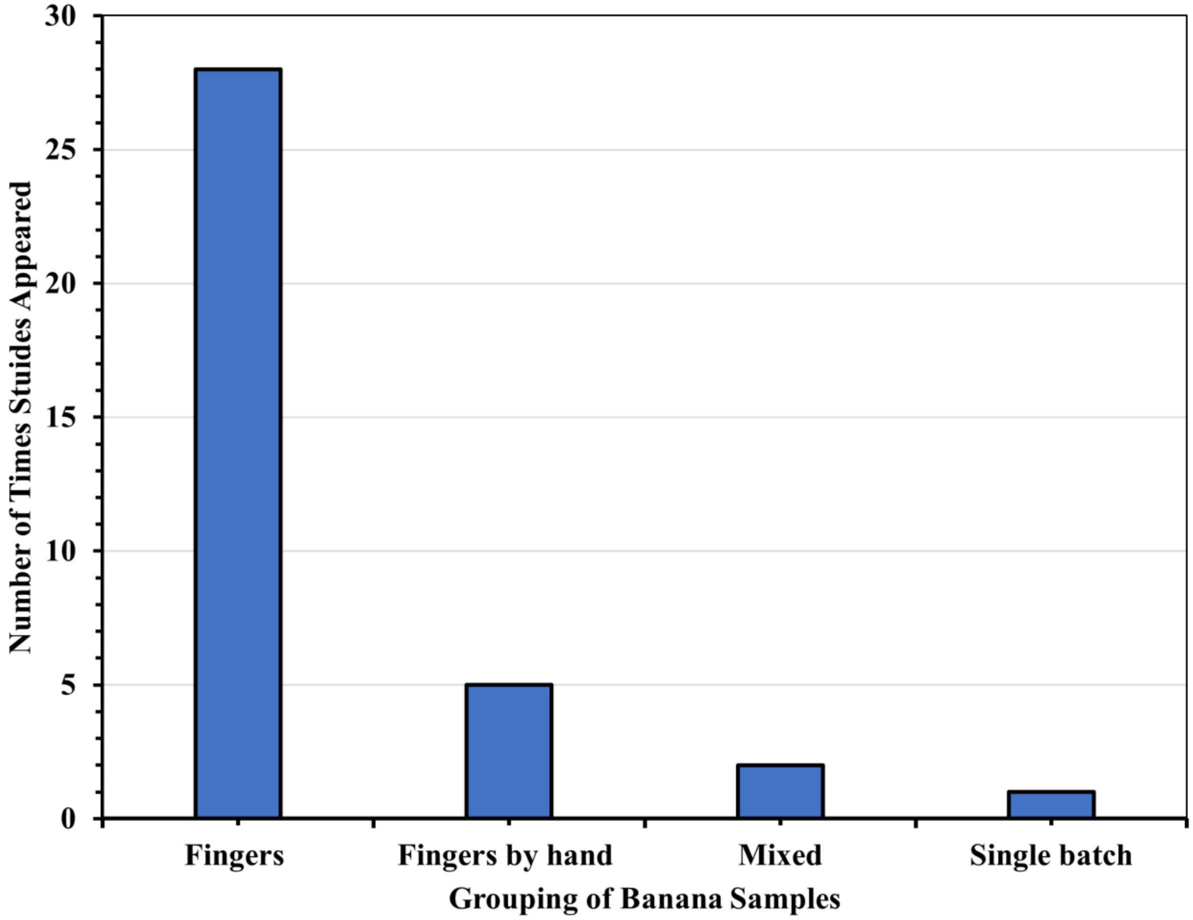

Figure S2. Grouping of banana samples used for detecting banana ripeness: single batch, bunch, mixed, fingers by hand, fingers, and Not Available (NA).

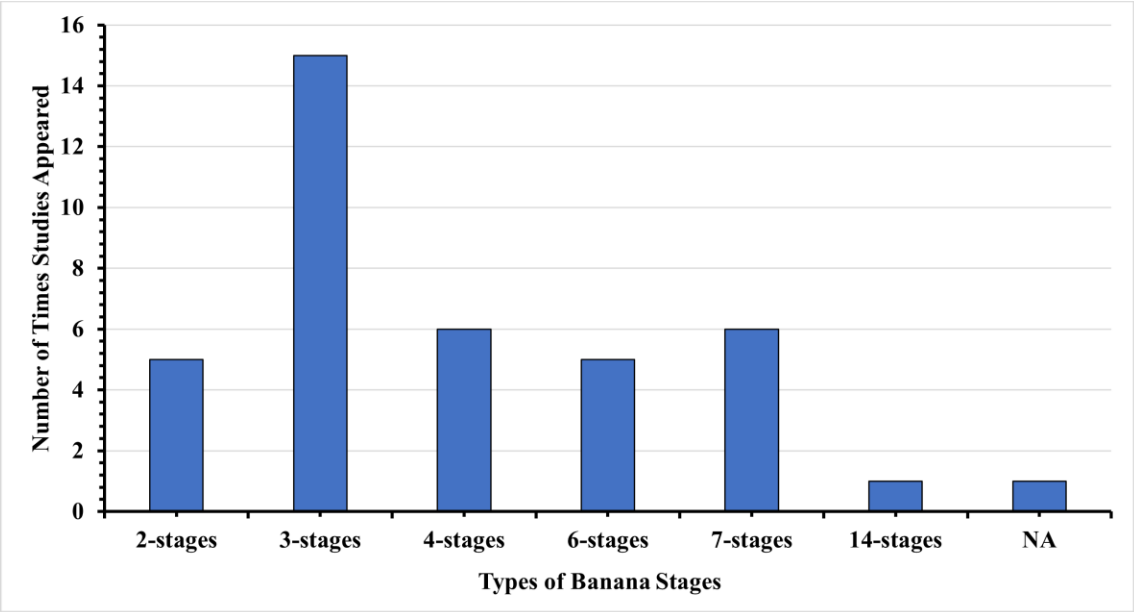

Figure S3. Types of different stages used for the detection of banana ripeness studies
